# Perceived Positive and Negative Life Changes in Testicular Cancer Survivors

**DOI:** 10.3390/medicina57090993

**Published:** 2021-09-20

**Authors:** Sigrun Vehling, Karin Oechsle, Michael Hartmann, Carsten Bokemeyer, Anja Mehnert-Theuerkauf

**Affiliations:** 1 Department of Medical Psychology, University Medical Center Hamburg-Eppendorf, 20246 Hamburg, Germany; 2 Department of Oncology, Hematology and Bone Marrow Transplantation with Section of Pneumology, University Medical Center Hamburg-Eppendorf, 20246 Hamburg, Germany; kaoechsl@uke.de (K.O.); cbokemeyer@uke.de (C.B.); 3 Department of Urology, University Medical Center Hamburg-Eppendorf, 20246 Hamburg, Germany; tschernigiw@gmx.de; 4 Department of Medical Psychology and Medical Sociology, University Medical Center Leipzig, 04103 Leipzig, Germany; anja.mehnert@medizin.uni-leipzig.de

**Keywords:** testicular cancer, perceived positive and negative life change, anxiety, depression, survivorship, posttraumatic growth

## Abstract

*Background and objectives:* Despite a generally good prognosis, testicular cancer can be a life-altering event. We explored perceived positive and negative life changes after testicular cancer in terms of frequency, demographic and disease-related predictors, and associations with depression and anxiety. *Materials and methods:* All testicular cancer survivors receiving follow-up care at two specialized outpatient treatment facilities were approached at follow-up visits or via mail. We assessed a total of *N* = 164 patients (66% participation rate, mean time since diagnosis: 11.6 years, SD = 7.4) by the Posttraumatic Growth Inventory (PTGI, modified version assessing positive and negative changes for each of 21 items), Patient-Health-Questionnaire-9 (PHQ-9), and Generalized-Anxiety-Disorder-Scale-7 (GAD-7). We conducted controlled multivariate regression analyses. *Results:* Most survivors (87%) reported at least one positive change (mean number: 7.2, SD = 5.0, possible range: 0–21). The most frequent perceived positive changes were greater appreciation of life (62%), changed priorities in life (62%), and ability rely on others (51%). At least one negative change was perceived by 33% (mean number of changes: 1.1, SD = 2.5). Negative changes were most frequent for decreases in self-reliance (14%), personal strength (11%), and ability to express emotions (9%). A higher socioeconomic status was associated with more positive changes (β = 0.25, 95% CI 0.08 to 0.42); no other association with demographic and disease-related predictors emerged. While positive life changes were not associated with depression (β = −0.05, 95% CI −0.17 to 0.07) and anxiety (β = 0.00, 95% CI −0.13 to 0.13), more negative life changes were significantly associated with higher depression (β = 0.15, 95% CI −0.03 to 0.27) and anxiety (β = 0.23, 95% CI 0.11 to 0.36). There was no significant interaction of positive and negative changes on depression or anxiety. *Conclusions:* Although positive life changes after testicular cancer are common, a significant number of survivors perceive negative changes in life domains that have been primarily investigated in terms of personal growth. Early identification of and psychosocial support for patients who perceive predominantly negative changes may contribute to prevention of prolonged symptoms of anxiety and depression.

## 1. Introduction

Many survivors report that, despite its stressfulness, the diagnosis and treatment of cancer has also had positive consequences on their lives [1,2]. Frequent areas of such perceived positive changes are one’s sense of relatedness with others, appreciation of life, and ability to cope with challenges in life [3], often accompanied by a sense of personal growth as a result of having gone through cancer-related adversity.

From a conceptual perspective, the perceived positive changes in the face of a stressful life event can be understood as an outcome of successful psychological adaptation efforts. For example, meaning-making processes can promote the integration of stressful experiences with previously held beliefs and life goals [4]. Under the threat of losing one’s life, close relationships, autonomy, and health due to a serious illness, survivors may reevaluate what is important in their lives and make fundamental changes to their private and vocational relationships. At the same time, perceived positive changes have been understood as adaptive illusions that help individuals to cope with the losses and negative consequences of a critical life event by positive reappraisal and benefit-finding [5,6].

Despite a generally good prognosis, the diagnosis and treatment of testicular cancer confronts individuals at an often earlier-than-expected life phase with long-term side effects of chemotherapy treatment, sexual dysfunction, a changed body image, hospitalization, and potential death [7]. Although perceived positive life changes and cancer-related growth have been documented in this population [8,9], many testicular cancer survivors find it difficult to return to normality, and experience ongoing negative consequences for their quality of life and increased levels of psychological distress [10,11]. To our knowledge, no previous quantitative study has analyzed the extent and frequency of domains of perceived life changes in testicular cancer survivors so far.

Earlier studies show no or only small associations of perceived positive life changes and post-traumatic growth with lower psychological distress, including depression and anxiety, while negative changes were highly correlated with worse adjustment outcomes [12,13]. This finding may relate to the different psychological underpinnings of perceived positive change, but also to assessment and methodological issues [5]. Researchers have thus turned away from the exclusive study of positive personality change after stressful life events, and increasingly focus on coexisting positive and negative changes in domains of potential personal growth [13,14]. In this regard, modifications of assessment procedures have been proposed which allow individuals to rate positive *or* negative changes in each life domain covered by the particular instrument [15]. Such modified methods also allow for the study of interaction effects of perceived positive and negative changes on adaptation outcomes. A higher level of positive change may buffer the detrimental effect of perceived negative changes [13]. According to this idea, distress may be lowest in individuals with high positive and low to medium negative changes, and highest in individuals with high negative and little or no positive changes.

In the present study, we aimed to investigate

(1) the frequency and extent of perceived positive and negative life changes in survivors of testicular cancer,
(2) the extent to which sociodemographic and disease-related variables are associated with perceived positive and negative changes,
(3) the association of positive and negative life changes with depression and anxiety, and
(4) whether positive life changes moderated the association of negative life changes with depression and anxiety.

## 2. Methods

### 2.1. Participants and Procedures

In this cross-sectional study, we enrolled all adult male testicular germ cell tumor survivors receiving routine follow-up care at the uro-oncological outpatient ward of the University Medical Center within the University Cancer Center Hamburg and a specialized private practice in Hamburg, Germany over one year. Eligible patients had completed anti-tumor treatment for at least 12 months prior without any further evidence of disease. Exclusion criteria were severe cognitive or physical impairment and insufficient proficiency in German to complete questionnaires and provide written informed consent. All patients who fulfilled the study inclusion criteria were contacted by their treating germ cell tumor specialist either in a personal conversation or via mail and were asked to complete a set of questionnaires. Patients provided written informed consent prior to study inclusion. The study was approved by the research ethics committee of the medical association in Hamburg, Germany (reference number PV4171).

### 2.2. Measures

Demographic data were collected from patients using a standardized self-report questionnaire. Disease-related characteristics were obtained from medical charts.

We assessed perceived positive and negative life changes using a modified version of the *Posttraumatic Growth Inventory (PTGI)* [16]. The original PTGI asks individuals to rate the extent to which life changes occurred as a result of a specific stressful event, which is cancer diagnosis in the present study. Its 21 items belong to five subscales: new possibilities, relating to others, personal strength, spiritual change, and appreciation of life. Following the suggestion of Tennen and Affleck [15], we replaced each original item by its analogous current standing version (e.g., “I have a greater sense of closeness with others” was replaced by “I have a sense of closeness with others”). Perceived positive or negative life changes with respect to each current standing item were assessed by a separate change item (“This has changed due to my illness”). Response options for change items were −2 = high negative change, −1 = low negative change, 0 = no change, +1 = low positive change, +2 = high positive change. The *positive change count* refers to the total number of positive changes and may range from 0 to 21. The *total positive change score* refers to the sum of positive change ratings and may range from 0 to 42. The procedure is analogous for *negative change count* and *total positive change score*. Higher scores indicate higher positive or negative change.

We assessed depression using the Patient Health Questionnaire-9 (PHQ-9) [17]. The PHQ-9 is a self-report measure of depression according to DSM-IV/V criteria on a scale from 0 (not at all) to 3 (nearly every day). Total scores may range from 0 to 27. Scores ≥10 indicate at least moderate depression.

We assessed anxiety with the Generalized Anxiety Disorder Screener-7 (GAD-7) [18]. The scale measures the frequency of symptoms of generalized anxiety disorder during the past two weeks according to DSM-IV/V criteria on a scale from 0 (not at all) to 3 (nearly every day). GAD-7 scores range from 0 to 21. Scores ≥10 indicate at least moderate anxiety.

We assessed physical symptom burden with the self-report Memorial Symptom Assessment Scale—Short Form (MSAS-SF) [19]. The scale assesses the presence of 28 common physical symptoms in patients with cancer over the past week. The symptom count refers to the total number of symptoms reported and may range from 0 to 28.

We calculated the multidimensional socioeconomic status index (SES) as an aggregate score of school and professional education level, occupational status, and income. Calculation followed the standardized procedure applied by the German Health Survey [20]. SES scores range from 3 to 21. A low SES refers to scores ≤8, a middle SES to scores between 9 and 14, and a high SES to scores ≥15.

### 2.3. Statistical Analysis

We used R version 4.1.0 for all analyses [21]. We calculated descriptive statistics including means, standard deviations, and frequencies for demographic and disease-related characteristics, perceived positive and negative life changes, depression, and anxiety. Group differences were calculated by *t*-tests and χ²-tests. Cohen’s d was used as effect size for group differences. We calculated Pearson correlations among predictor and dependent variables to study bivariate correlations.

To analyze the association of positive and negative life change with depression and anxiety, we conducted a multiple linear regression analysis that controlled for age at diagnosis, cohabitation, children, socioeconomic status, time since diagnosis, metastatic disease, relapse, and physical symptom count. To test whether negative life change moderated the association of positive life change with depression and anxiety, we added an interaction term as a predictor to the regression model in a subsequent step. The interaction term was calculated by multiplying the standardized values of positive and negative life change scores.

## 3. Results

### 3.1. Sample Characteristics and Frequency of Perceived Positive and Negative Life Changes

Of the 255 eligible patients approached, 61.1% (*N* = 164) completed the questionnaire. Participants and non-participants did not differ in terms of age (d = 0.24, *p* = 0.18), years since diagnosis (d = 0.16, *p* = 0.39), and presence of metastases at first diagnosis (d = 0.00, *p* = 0.98). Table 1 shows demographic and disease-related characteristics as well as descriptive information for dependent variables.

Survivors reported a mean number of 7.2 (SD = 5.0) positive changes out of 21 possible positive changes (Table 2). Positive changes in at least one life domain after testicular cancer were reported by 87% of the survivors. As shown in Table 3, patients most frequently experienced positive changes in the following domains: appreciation of life in general (62%) and of each day (52%), knowing what is important in life (62%), and perceived ability to accept needing (43%) and to count on others in times of trouble (51%). Negative changes were remarkably less frequent, with 33% reporting at least one negative change after testicular cancer. The mean number of negative changes was M = 1.1 (SD = 2.5). Patients reported negative changes most frequently for their sense of self-reliance (13%), personal strength (11%), and ability to express emotions (9%).

Table 4 shows the co-occurrence of subgroups reporting no, low, and high numbers of positive and negative changes at the same time. *N* = 19 (11.6%) survivors reported no changes at all. *N* = 51 (31%) reported both positive and negative changes. This corresponded to 94% of those with negative changes reporting also positive change, and 36% of those with positive changes reporting simultaneous negative changes.

The mean total positive change score, which accounts for the total number (0–21) and degree of positive and negative changes perceived in each domain (0–2), was 9.6 (SD = 6.7, range 0–27, possible range 0–42). The mean total negative change score was 1.5 (SD = 4.2, range = 0–38). The correlation of positive and negative change scores was r = −0.12 (*p* = 0.39).

### 3.2. Sociodemographic and Disease-Related Predictors of Perceived Positive and Negative Life Changes

All following analyses were performed using the total positive and negative change scores. Table 5 shows that in the multivariate model combining all predictors, only a higher socioeconomic status was significantly associated with a higher level of perceived positive changes. None of the other assessed demographic and disease-related variables showed any significant association with positive or negative changes.

### 3.3. Association of Perceived Positive and Negative Life Changes with Depression and Anxiety

Table 6 shows bivariate correlations between positive and negative life changes and outcome variables. Table 7 shows regression weights for the association of positive and negative life changes with depression and anxiety, controlled for demographic and disease-related variables. A higher level of positive life changes was not associated with depression (β = −0.05, 95% CI −0.17 to 0.07) or anxiety levels (β = 0.00, 95% CI −0.13 to 0.13). Yet a higher level of negative changes was significantly associated with higher levels of depression (β = 0.15, 95% CI −0.03 to 0.27) and anxiety (β = 0.23, 95% CI 0.11 to 0.36), even when the impact of age, gender, physical symptoms, and other important confounders was controlled. There was no significant interaction of positive and negative life changes on outcome variables when confounders were controlled.

## 4. Discussion

The present study analyzed perceived positive and negative life changes among 164 testicular cancer survivors (mean age at diagnosis: 33 years, mean time since diagnosis: 21 years, metastatic disease: 48%). We used a modified version of the posttraumatic growth inventory to assess positive and negative subjective life changes across 21 domains. While almost two thirds of participants reported positive changes in six or more life domains, one third experienced at least one negative change. Of those experiencing negative changes, 94% experienced simultaneous positive change.

The high percentage of survivors reporting positive life changes is consistent with studies using the original posttraumatic growth inventory in cancer patients [22]. Qualitative studies in testicular cancer survivors have documented, consistent with the present results, a variety of perceived positive outcomes [23,24]. The present lack of a relationship between a higher level of positive change and lower psychological distress is in line with earlier studies [13,25]. One possible explanation is that the perception and report of positive changes may reflect efforts to cope with a psychologically demanding situation and not necessarily actual personality change. Positive reappraisal of negative implications is known to play a central role in adaptation to severe illness [26]. This is also supported by findings in testicular cancer patients that positive changes were associated with effective goal adjustment efforts and approach-oriented coping [23]. A study of Park et al. [27] has further shown that positive changes reported by young and middle aged survivors were not directly associated with actual changes.

Although negative consequences after testicular cancer have been mostly studied in terms of long-term side-effects, our results suggest that perceived negative changes in psychological domains play an important role for the subjective experience of survivors. Consistent with the present results, testicular cancer survivors were found to experience the diagnosis as a disruptive event that led to an increased sense of vulnerability and impaired sense of connectedness with significant others [28]. In an interview study, many young adult cancer survivors also reported coexisting positive and negative changes [29]. The surprising result that negative changes were, contrary to studies in other cancer populations [13,30], not associated with younger age, metastasized disease, shorter time since diagnosis, or physical symptom burden in the present study, may further strengthen the relevance of psychological resources for coping with testicular cancer over objective prognostic and treatment-related factors [24].

The significant contribution of perceived negative life changes to depression and anxiety beyond physical symptom burden and other established confounders provides further support for the idea that a higher number of negative changes might increase the vulnerability for prolonged psychological distress [13]. That negative changes were most frequent in areas of self-reliance and emotional expression may suggest increased coping demands for patients whose regulation of self-worth is closely tied to physical or emotional strength, which may relate to avoiding expression of emotional states and need for independence in close relationships. Clinicians may ask survivors whether they feel distressed by an ongoing sense of weakness or disconnect from significant others in order to explore a potential need for extended psychosocial support beyond routine survivorship care [7]. Such support may provide an in-depth elaboration of life areas potentially affected by testicular cancer including the interconnection of fertility, sexuality, self-worth and relationships [28]. It is important to note that patients reporting a high number of negative changes may have developed distorted negative perceptions as a symptom of clinical depression, requiring treatment according to guidelines.

Our study is limited by its cross-sectional design, a rather small sample size, and high heterogeneity with respect to age at diagnosis and time since diagnosis. Moreover, our assessment did not cover the possibility of simultaneous positive and negative change in the same domain, i.e., survivors might experience an increased sense of reliability of certain others (positive change), but feel disappointed by others (negative change). The newly developed Posttraumatic Growth and Posttraumatic Depreciation Inventory-Expanded version (PTGDI-X, [14]) offers a validated assessment of positive and negative perceived changes after stressful life events.

## 5. Conclusions

Although positive life changes were common, one third of survivors perceived negative life changes after testicular cancer. Thus, a significant subgroup reports negative changes in life domains that are usually investigated in terms of potential personal growth after cancer. Domains with negative changes included depreciated feelings of personal strength, self-reliance, and ability for emotional expression. A higher number of negative changes may increase the risk for prolonged psychological distress. Objective medical factors were not associated with the perception of positive or negative changes. Future longitudinal studies should therefore investigate the specific psychological processes that lead to the perception of positive and negative illness-related changes over the course of treatment and survivorship of testicular cancer. Early identification of patients who perceive predominantly negative changes may contribute to prevention of prolonged symptoms of anxiety and depression.

## Figures and Tables

**Table 1:** Demographic and disease related sample characteristics and descriptive statistics for dependent variables (*N* = 164).

Variable	*N*	%
**Age**, mean (SD, range)	44.4 (9.6, 24–77)	
**Age at diagnosis**, mean (SD, range)	32.8 (9.5)	
**Cohabiting**	136	83
**Children**	86	52
**Education**		
Elementary school (8–9 years)	17	10
Junior high school (10 years)	40	25
High school (12–13 years)	23	14
University	84	51
**Socioeconomic status index** ^a^, mean (SD)	13.0 (3.5)	
Low	24	16
Middle	77	51
High	51	34
**Time since diagnosis**, mean (SD)	11.6 (7.4)	
1–5 years	50	30
6–10 years	34	21
11–15 years	33	20
16–20 years	27	17
≥21 years	20	12
**Disease stage**		
Localized disease	85	52
Metastatic disease	79	48
**Disease phase**		
First disease	136	83
Relapse	28	17
**Medical treatments**		
Chemotherapy	124	76
Surgery	160	98
Radiotherapy	38	23
**Physical symptom count**, mean (SD) ^b^	4.5 (4.3)	
**Depression**, mean (SD) ^c^	3.6 (3.6)	
Low to mild	151	92.1
Moderate to high	13	7.9
**Anxiety**, mean (SD) ^d^	3.3 (3.3)	
Low to mild	154	93.9
Moderate to high	10	6.1

^a^ Possible range: 3–21. ^b^ Possible range: 0–28. ^c^ Possible range: 0–27. ^d^ Possible range: 0–21.

**Table 2:** Number of perceived positive and negative life changes for 21 life domains (*N* = 164).

	Number Of Positive Life Changes		Number of Negative Life Changes	
	*N*	%	*N*	%
0 (no change)	22	13.4	110	67.1
1–5 domains	43	17.7	46	28.0
6–10 domains	56	42.7	6	3.7
11–15 domains	32	19.5	1	0.6
16–21 domains	11	6.7	1	0.6
Mean (SD)	7.2 (5.0)		1.1 (2.5)	

**Table 3:** Frequency of patients reporting positive and negative change for each life domain (*N* = 164).

Domains of Positive Life Changes			Domains of Negative Life Changes		
	*N*	%		*N*	%
Priorities about what is important	102	62.2	Self-reliance	22	13.4
Appreciation for value of life	101	61.6	Personal strength	18	11.0
Appreciate each day	85	51.8	Express emotions	14	8.5
Count on people in times of trouble	83	50.6	Understand spiritual matters	13	7.9
Accept needing others	70	42.7	Strength of religious faith	10	6.1
Do better things with life	67	40.9	Handle difficulties	10	6.1
Accept the way things work out	67	40.9	Priorities about what is important	9	5.5
Handle difficulties	66	40.2	Establish new path for life	8	4.9
Closeness with others	63	38.4	Do better things with life	8	4.9
Compassion for others	60	35.9	Develop new interests	7	4.3
Put effort into relationships	59	36.0	Appreciation for value of life	7	4.3
Change things that need changing	50	30.5	Change things that need changing	7	4.3
Personal strength	50	30.5	Goodness of others	7	4.3
Express emotions	46	28.0	Count on people in times of trouble	6	3.7
Establish new path for life	46	28.0	Accept the way things work out	5	3.0
Develop new interests	41	25.0	Compassion for others	5	3.0
Self-reliance	41	25.0	Put effort into relationships	5	3.0
Goodness of others	36	22.0	Accept needing others	5	3.0
New opportunities available	22	13.4	New opportunities available	4	2.4
Understand spiritual matters	22	13.4	Closeness with others	4	2.4
Strength of religious faith	17	10.4	Appreciate each day	1	0.6

**Table 4:** Co-occurrence of positive and negative life changes (*N* = 164).

	Number of Positive Life Changes		
Number of Negative Life Changes	No Change (0)	Low (1–5)	High (6–21)
No change (0)	19 (11.6%)	27 (16.5%)	64 (39.0%)
Low (1–5)	2 (1.2%)	12 (7.3%)	32 (19.5%)
High (6–21)	1 (0.6%)	4 (2.4%)	3 (1.8%)

**Table 5:** Multiple linear regression model analyzing predictors of positive and negative life change scores (*N* = 164).

	Positive Life Change			Negative Life Change		
	β ^a^	95% CI	*p*	β ^a^	95% CI	*p*
Age at diagnosis	−0.13	−0.31 to 0.06	0.17	0.17	−0.02 to 0.35	0.08
Cohabitation ^b^	−0.09	−0.26 to 0.08	0.31	−0.06	−0.24 to 0.11	0.49
Children ^b^	0.09	−0.08 to 0.27	0.28	−0.01	−0.19 to 0.17	0.92
Socioeconomic status	**0.26**	0.09 to 0.44	0.003	0.02	−0.16 to 0.20	0.84
Time since diagnosis (>10 years vs. ≤10 years)	0.08	−0.10 to 0.25	0.40	0.06	−0.12 to 0.25	0.52
Metastatic vs. localized disease	0.09	−0.08 to 0.26	0.27	0.06	−0.12 to 0.23	0.52
Physical symptom count	0.07	−0.09 to 0.24	0.38	0.11	−0.06 to 0.29	0.21

^a^ Standardized regression coefficients. ^b^ 0 = No, 1 = Yes; Total variation accounted for: Positive life change: adj. R² = 0.07, Negative life change: R² = 0.00. Significant β-values are printed bold.

**Table 6:** Intercorrelations of positive and negative life changes, depression, and anxiety (*N* = 164).

	Negative Life Change ^a^	Depression	Anxiety
Positive life change ^a^	−0.12	−0.07	0.00
Negative life change ^a^	-	0.50 ***	0.50 ***
Depression	-	-	0.77 ***

^a^ Total change score. *** *p* ≤ 0.001.

**Table 7:** Impact of positive and negative change on depression and anxiety (*N* = 164).

	Depression			Anxiety		
	β ^a^	95% CI	*p*	β ^a^	95% CI	*p*
*Model 1: Positive life change*						
Age at diagnosis	−0.04	−0.17 to 0.09	0.57	**−0.17**	−0.31 to −0.03	0.02
Cohabitation ^b^	**−0.16**	−0.29 to −0.04	0.009	−0.05	−0.18 to 0.09	0.50
Children ^b^	**0.13**	0.01 to 0.25	0.03	**0.17**	0.04 to 0.30	0.01
Socioeconomic status	−0.01	−0.14 to 0.11	0.84	0.07	−0.06 to 0.21	0.28
Time since diagnosis (>10 years vs. ≤10 years)	−0.06	−0.19 to 0.06	0.33	−0.13	−0.27 to 0.00	0.06
Metastatic vs. localized disease	−0.10	−0.22 to 0.02	0.11	**−0.16**	−0.29 to −0.03	0.02
Physical symptom count	**0.71**	0.59 to 0.83	<0.001	**0.68**	0.55 to 0.81	<0.001
Positive life change	−0.05	−0.17 to 0.07	0.40	0.00	−0.13 to 0.13	0.97
*Model 2: Negative life change*						
Age at diagnosis	−0.02	−0.15 to 0.10	0.70	**−0.16**	−0.29 to −0.02	0.02
Cohabitation ^b^	**−0.14**	−0.26 to −0.02	0.02	−0.02	−0.14 to 0.11	0.79
Children ^b^	**0.13**	0.01 to 0.25	0.03	**0.18**	0.05 to 0.30	0.01
Socioeconomic status	−0.01	−0.13 to 0.11	0.88	0.10	−0.02 to 0.23	0.11
Time since diagnosis (>10 years vs. ≤10 years)	−0.08	−0.21 to 0.04	0.20	**−0.16**	−0.29 to −0.03	0.02
Metastatic vs. localized disease	−0.010	−0.22 to 0.02	0.10	**−0.16**	−0.28 to −0.03	0.01
Physical symptom count	**0.66**	0.54 to 0.79	<0.001	**0.61**	0.48 to 0.74	<0.001
Negative life change	**0.15**	−0.03 to 0.27	0.02	**0.23**	0.11 to 0.36	<0.001
*Model 3: Interaction of positive and negative life change*						
Age at diagnosis	−0.03	−0.15 to 0.10	0.67	**−0.16**	−0.29 to −0.03	0.02
Cohabitation ^b^	**−0.14**	−0.27 to −0.02	0.02	−0.02	−0.15 to 0.10	0.72
Children ^b^	**0.13**	0.01 to 0.26	0.03	**0.19**	0.06 to 0.31	0.004
Socioeconomic status	0.00	−0.13 to 0.13	0.99	0.11	−0.03 to 0.24	0.11
Time since diagnosis (>10 years vs. ≤10 years)	−0.08	−0.20 to 0.05	0.22	**−0.16**	−0.29 to −0.03	0.02
Metastatic vs. localized disease	−0.10	−0.22 to 0.02	0.11	**−0.15**	−0.27 to −0.03	0.02
Physical symptom count	**0.67**	0.55 to 0.79	<0.001	**0.60**	0.47 to 0.73	<0.001
Positive life change	−0.04	−0.16 to 0.08	0.44	0.00	−0.12 to 0.13	0.47
Negative life change	0.16	0.03 to 0.30	00.16	**0.20**	0.05 to 0.34	<0.001
Positive × negative life change (interaction effect)	0.03	−0.11 to 0.16	0.69	−0.11	−0.25 to 0.04	0.14

^a^ Standardized regression coefficients. ^b^ 0 = No, 1 = Yes. Significant β-values are printed bold.

## Data Availability

The data that support the findings of this study are available from the corresponding author upon reasonable request.

## Abbreviations

M	Mean
SD	Standard deviation
CI	Confidence interval
PTGI	Posttraumatic Growth Inventory
PHQ-9	Patient-Health-Questionnaire-9
GAD-7	Generalized-Anxiety-Disorder-Scale-7
MSAS-SF	Memorial Symptom Assessment Scale—Short Form
SES	Socioeconomic status index
